# Unravelling the Portuguese Coastal and Transitional Waters’ Microbial Resistome as a Biomarker of Differential Anthropogenic Impact

**DOI:** 10.3390/toxics10100613

**Published:** 2022-10-15

**Authors:** Bernardo Duarte, Andreia Figueiredo, Patrício Ramalhosa, João Canning-Clode, Isabel Caçador, Vanessa F. Fonseca

**Affiliations:** 1 MARE—Marine and Environmental Sciences Centre and ARNET—Aquatic Research Infrastructure Network Associated Laboratory, Faculdade de Ciências da Universidade de Lisboa, Campo Grande, 1749-016 Lisbon, Portugal; 2 Departamento de Biologia Vegetal, Faculdade de Ciências da Universidade de Lisboa, Campo Grande, 1749-016 Lisboa, Portugal; 3 BioISI—Biosystems and Integrative Sciences Institute, Plant Functional Genomics Group, Departamento de Biologia Vegetal, Faculdade de Ciências da Universidade de Lisboa, Campo Grande, 1749-016 Lisboa, Portugal; 4 MARE—Marine and Environmental Sciences Centre and ARNET—Aquatic Research Infrastructure Network Associated Laboratory, Agência Regional para o Desenvolvimento da Investigação Tecnologia e Inovação (ARDITI), Edifício Madeira Tecnopolo Piso 0, Caminho da Penteada, 9020-105 Funchal, Portugal; 5 OOM—Oceanic Observatory of Madeira, Agência Regional para o Desenvolvimento da Investigação Tecnologia e Inovação (ARDITI), Edifício Madeira Tecnopolo Piso 0, Caminho da Penteada, 9020-105 Funchal, Portugal; 6 Smithsonian Environmental Research Center, 647 Contees Wharf Road, Edgewater, MD 21037, USA; 7 Departamento de Biologia Animal, Faculdade de Ciências da Universidade de Lisboa, Campo Grande, 1749-016 Lisboa, Portugal

**Keywords:** ocean metagenomes, resistance genes, anthropogenic pollution, resistome

## Abstract

Portugal mainland and Atlantic archipelagos (Madeira and Azores) provide a wide array of coastal ecosystems with varying typology and degrees of human pressure, which shape the microbial communities thriving in these habitats, leading to the development of microbial resistance traits. The samples collected on the Portuguese northeast Atlantic coast waters show an unequivocal prevalence of Bacteria over Archaea with a high prevalence of Proteobacteria, Cyanobacteria, Bacteroidetes and Actinobacteria. Several taxa, such as the *Vibrio* genus, showed significant correlations with anthropogenic pollution. These anthropogenic pressures, along with the differences in species diversity among the surveyed sites, lead to observed differences in the presence and resistance-related sequences’ abundance (set of all metal and antibiotic resistant genes and their precursors in pathogenic and non-pathogenic bacteria). Gene ontology terms such as antibiotic resistance, redox regulation and oxidative stress response were prevalent. A higher number of significant correlations were found between the abundance of resistance-related sequences and pollution, inorganic pressures and density of nearby population centres when compared to the number of significant correlations between taxa abundance at different phylogenetic levels and the same environmental traits. This points towards predominance of the environmental conditions over the sequence abundance rather than the taxa abundance. Our data suggest that the whole resistome profile can provide more relevant or integrative answers in terms of anthropogenic disturbance of the environment, either as a whole or grouped in gene ontology groups, appearing as a promising tool for impact assessment studies which, due to the ubiquity of the sequences across microbes, can be surveyed independently of the taxa present in the samples.

## 1. Introduction

Coastal and transitional ecosystems (estuaries and coastal lagoons) are among the most studied systems in terms of anthropogenic-driven impacts (e.g., [1]). In the last decades, a significant effort has been made by competent authorities to reduce the anthropogenic footprint left on these ecosystems, with an evident reduction in the sediment metal burden from the 1960s to the present [2]. Despite this reduction in the anthropogenic input of classic contaminants, these are still frequently persistent in the environment, with the added threat of newly emerging contaminants such as microplastics, pharmaceuticals and personal care products [3]. The prevalence and consequent significant mobility of these contaminants in the ecosystems [4] can even be detected in remote uninhabited locations, with an unexpected presence in key trophic compartments such as the planktonic microbial community [5,6]. Beyond the necessary environmental risk assessment (ERA) approaches, to evaluate the possible impacts of these contaminants in the different biotic compartments [7], it is also essential to understand how these xenobiotics interact with the organisms and what are the molecular implications of these interactions. These biochemical and molecular traits that suffer changes from their exposure to a certain xenobiotic or contaminant cocktail (also known as exposome) can be used as contamination biomarkers for impact assessment studies.

Several sets of biomarkers are known to respond efficiently to external contamination in several marine biotic compartments, such as phytoplankton [8,9], plants [10,11,12], macroalgae [13], macroinvertebrates [14] and fishes [15]. As for bacteria, a new set of biomarkers can be of added value, highlighted in recent studies that feature the great value of this microbial, often disregarded, black box for ecological quality assessment purposes [16,17]. Some authors include microbial communities into taxonomic classification indexes, with bacterial assemblages complementing the information provided by benthic metazoan communities as indicators of human-induced impacts [16]. On the other hand, DNA sequencing may also offer insight into ecosystem stress via changes in relative abundance or completeness of enzymatic pathways associated with chemical biodegradation or resistance [17]. Nevertheless, it is important to bear in mind that these are not mutually exclusive evaluations. In our point of view, changes in the community can imply changes in the community’s metabolic traits, but metabolic changes do not necessarily imply bacterial assemblages’ changes in taxonomic terms.

The interaction of these microorganisms with classic and emerging contaminants induces expectable biochemical stress levels and allows the development of resistance traits. The term resistome was proposed by D’Costa et al. [18] to encompass the set of all antibiotic resistance genes and their precursors in pathogenic and non-pathogenic bacteria. Since then, several studies have been conducted in soils [19], wastewater metacommunities [20] and across metagenome libraries [21]. Nevertheless, these studies mainly focused on antibiotic-resistance genes. However, the mechanisms of resistance/tolerance to biocides and heavy metals may also promote resistance/tolerance to antibiotics and have recently been included in the definition of resistome [19,22,23,24]. More recently, other contaminants have been suggested to induce resistance to other xenobiotics, pointing out the importance to include cross-tolerance mechanisms in resistome assessments. Silva et al. [25] results indicate that the prevalence of antibiotic resistance genes is directly connected to heavy metal contamination along a riverine gradient. In coastal and transitional systems, the prevalence of several and diffuse anthropogenic pressures can thus lead to the development of different resistomes, namely in terms of antibiotic resistance genes (ARGs) and heavy metal resistance genes (HMRGs) [25]. It becomes, therefore, important to perform a wide scale evaluation of the presence of these ARGs and HMRGs.

In this context, large scale metagenomic efforts such as the ones developed in the MicroB3 project through the Ocean Sampling Day (OSD) initiative [26] or through the TARA Oceans project [27] represent a unique opportunity to cover significant parts of the marine realm and produce enormous amounts of metagenomic and metatranscriptomic data about the Earth’s oceans. This OSD initiative allowed for employing, on a global scale, a standardized procedure in every single sampling point, and the effort to sequence all the samples in a single laboratory created a genomic database of the ocean’s microbial life that can be used to disclose several unknown aspects of this hidden majority, the ocean microbiome [26].

In this sense, this work aimed to investigate how anthropogenic abiotic features may affect the microbial composition and the ARGs’ and HMRGs’ profiles in different sites sampled along the Portuguese mainland and the islands’ coastal and transitional systems using metagenomics.

## 2. Materials and Methods

### 2.1. Sample Collection and Environmental Data

**Figure 1:**
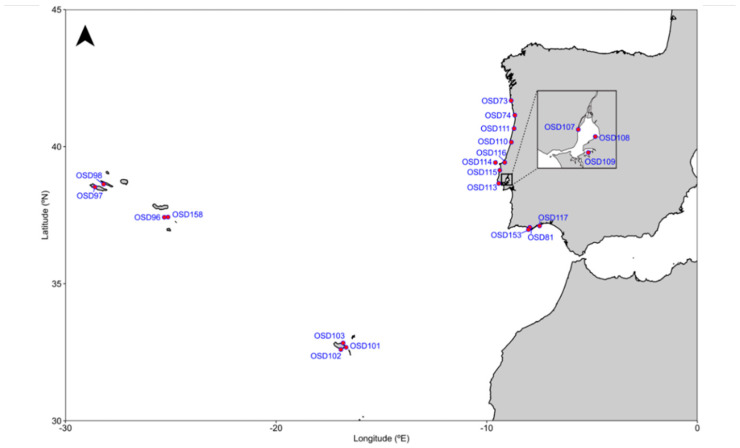
Surveyed sampling stations on the Portugal mainland and Madeira and Azores archipelagos.

Surface water samples from 21 marine locations (Figure 1) were collected on 21 June 2014 and transported, refrigerated with ice blocks, to the laboratory. Sampling sites included coastal beaches, estuarine areas, coastal lagoons, marinas and Atlantic islands’ coasts. Temperature and salinity data were obtained through direct measurements from in situ water sampling. At the laboratory, all samples were processed according to the MicroB3 Ocean Sampling Day filtration protocols (https://www.microb3.eu/sites/default/files/deliverables/MB3_D4_3_PU.pdf, accessed on August 2022). Samples were filtered with 0.22 μm pore size Sterivex filters (Merck Millipore), without prefiltration, and preserved at −80 °C. Filters were then shipped in dry ice to Bremen University for sequencing, as described below, within X days after sampling. Metadata (temperature and salinity) were obtained from https://github.com/MicroB3-IS/osd-analysis/wiki/Guide-to-OSD-2014-data (accessed on August 2022). Anthropogenic pressures (such as inorganic pressure, pollution, plume influence, shipping influence and nearby population) were derived from the Halpern database [28] and deposited in the PANGEA database [29], available for download at https://github.com/MicroB3-IS/osd-analysis/wiki/OSD-2014-environmental-data-csv-documentation (accessed on August 2022). Distance to the coast was calculated for each station using Rgdal and Rgeos packages and the coastline file available (http://www.naturalearthdata.com/downloads/10m-physical-vectors/10m-coastline/, accessed on August 2022) using the R-Studio Version 1.4.1717 software. Site metadata are presented in Table 1.

### 2.2. Metagenomic Sequencing and Bioinformatic Processing

All molecular processing steps were performed by the OSD team. The DNA was extracted using the Power Water isolation kit (MoBio, Carlsbad, CA, USA) following the manufacturer instructions. Amplification of the 16S rRNA gene was performed using the primer pair 515F (5′-GTGCCAGCMGCCGCGGTAA-3′) and 806R (5′-GGACTACHVGGGTWTCTAAT-3′), selective for both Archaea and Bacteria [30], which was designed as part of the Earth Microbiome Project [31]. The Illumina libraries were prepared using the Ovation Rapid DR Multiplex System 1–96 (NuGEN, link to protocol: https://owncloud.mpi-bremen.de/index.php/s/RDB4Jo0PAayg3qx?path=/2014/protocols, accessed on August 2022). Sequencing (2 × 250 paired end) was performed with Illumina technology MiSeq using V3 chemistry by the LGC genomics GmbH (Germany, http://www.lgcgroup.com/, accessed on August 2022).

Fasta files (http://mb3is.megx.net/osd-files?path=/2014/datasets/workable, accessed on August 2022) were provided by the OSD consortium. The raw files and pipeline process data have been deposited at EBI (https://www.ebi.ac.uk/metagenomics/studies/MGYS00000462#overview, accessed on August 2022). All subsequent sequence processing was performed with Mothur v 1.35.1 [32]. Reads were filtered to remove those shorter than 300 bp or containing ambiguities (N). Then, reads were aligned to SILVA seed release 123 alignments [33], corrected manually with the Geneious software v 7.1.7 [34]: gaps at the beginning and the end of sequences were deleted. Chimaeras were checked using Uchime v 4.2.40 [35] as implemented in Mothur. Data were trimmed to remove chloroplast sequence data. The datasets were pre-clustered using Mothur. After distance matrix calculation, the sequences were clustered using the Nearest Neighbour method, and Operational Taxonomic Units (OTUs) were built at 99% similarity. OTUs represented by only one sequence (singletons) were deleted. The OTUs were finally assigned using the Wang approach [36] and the PR^2^ database [37], available at https://doi.org/10.6084/m9.figshare.5913181 (accessed on August 2022), for which the Bacteria and Archaea sequences had been checked against the most recent taxonomy. Operational Taxonomic Units presenting bootstrap levels lower than 80% were not considered. Each OTU was linked to a reference sequence, and an OTU was assigned when the lowest taxonomic level (“Genus” level in PR^2^) differed from “unclassified”. In order to validate OTU assignment, megablast was performed against the GenBank database [38]. Relative abundances of each OUT were determined by dividing the number of hits of a specific OUT in a sample by the total number of hits of the same sample. For the resistome analysis, sequence signatures and Gene Ontology terms (GO terms) were assigned by the InterPro database [39] http://www.ebi.ac.uk/interpro/ (accessed on August 2022). The relative abundance of each InterPro protein signature was calculated by dividing the number of hits of a specific InterPro signature in a sample by the total number of resistance-related InterPro hits. Only resistance proteins or domains associated to resistance characteristics were considered for representation purposes.

Heatmaps were constructed using the *ggplot2 ()* package in R-Studio Version 1.4.1717. Spearman correlation coefficients and statistical significance between the fatty acid traits, index, exogenous concentration and growth trait values were computed using the *corrplot ()* package in R-Studio Version 1.4.1717. Principal Coordinates Analysis (PCoA) was preformed using the *vegan ()* package in R-Studio Version 1.4.1717.

## 3. Results

### 3.1. Coastal Archaea and Bacteria Taxonomic Diversity

Regarding the prokaryote kingdom diversity, all sampling stations showed a prevalence of Bacteria over Archaea. Bacteria comprised more than 99% of the prokaryote OTUs of the samples. As for Archaea, the OSD98 sampling station was the site with a higher Archaea relative abundance (0.64%), while the OSD114 station showed the lowest abundance (0.11%) of OTUs from this kingdom.

**Figure 2:**
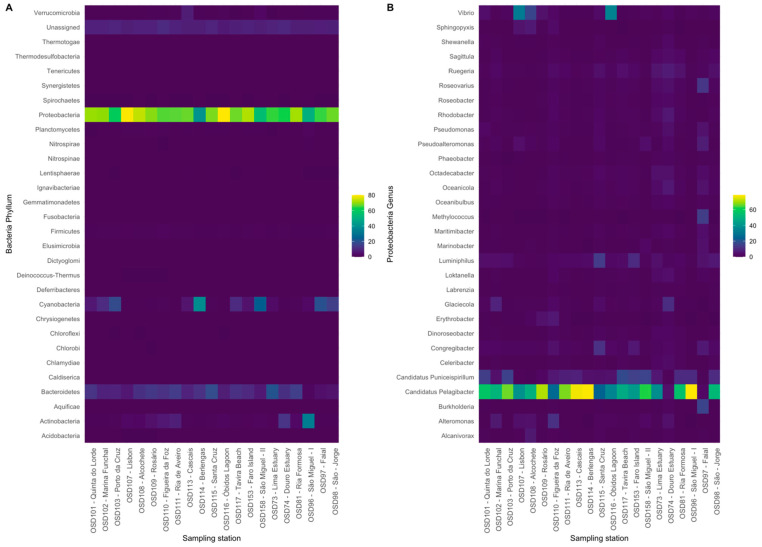
Bacteria phyla (**A**) and Proteobacteria genus (**B**) relative abundances in the surface water samples collected at the 21 sampling sites on the Portugal mainland and islands.

**Figure 3:**
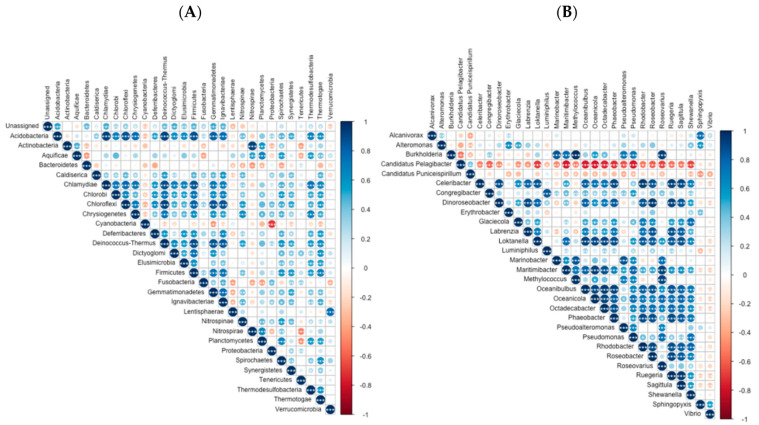
Spearman correlogram between the phyla (**A**) and Proteobacteria genus (**B**) relative abundances along with the surveyed surface water samples from the Portugal mainland and islands (asterisks denote significant Spearman correlations at * *p* < 0.05, ** *p* < 0.01 and *** *p* < 0.001).

Due to the complete dominance of Bacteria over Archaea, the abundance of Bacteria phyla was analysed (Figure 2A). Proteobacteria were the prevalent phylum in all samples, ranging from 80.2% in the sampling site OSD116 (Óbidos Lagoon) to 40.2% in OSD114 (Berlengas), having an average relative abundance over the whole set of sampling sites of 66.1% of all the bacteria phyla detected. The Cyanobacteria were the second most abundant phylum detected in the evaluated surface water samples. In fact, in the site with the lowest prevalence of Proteobacteria (40.2% in OSD114—Berlengas), Cyanobacteria prevailed. Nevertheless, very low Cyanobacteria relative abundances were also generally detected, with minimum values detected in OSD111 (0.27%) and presenting an average relative abundance across the surveyed samples of 7.9%. The phylum Bacteroidetes also presented relatively high abundances in the collected water samples, with values ranging from 20.3% (OSD73—Lima Estuary) to 2.16% (OSD96—São Miguel I) and an average relative abundance value of 10.5%. The fourth most abundant phylum assessed in the sample dataset was the Actinobacteria. This phylum presented a maximum abundance of 33.4% (OSD96—São Miguel I) and a minimum relative abundance value of 0.49% (OSD114—Berlengas) in the evaluated dataset, with an average relative abundance of 4.07%. The relative abundance of OTUs to which it was impossible to assign a specific phylum was always very low (Unassigned, 6.8 to 10.5%, average value = 8.14%). This interchange between phyla in the surveyed samples is evident in Figure 3A. Cyanobacteria’s and Bacteroidetes’ relative abundance presented a significant inverse correlation with almost all the phyla relative abundances. Actinobacteria showed highly dynamic trends, with significant direct correlations between their relative abundance and the abundances of the phyla Aquificae, Nitrospirae, Planctomycetes and Spirochaetes. On the other hand, inverse and significant correlations were found between Actinobacteria’s relative abundance and the prevalence of Bacteroidetes, Fusobacteria, Proteobacteria and Tenericutes.

**Figure 4:**
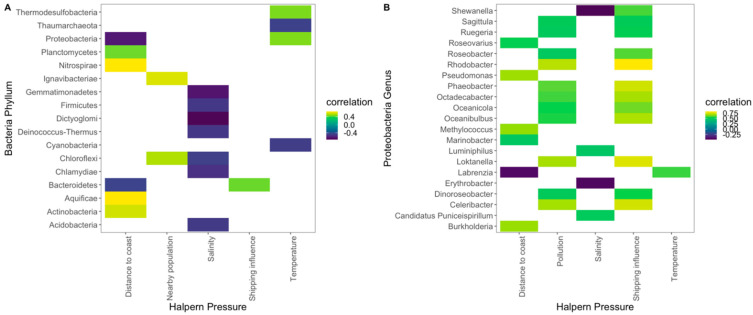
Significant (*p* < 0.05) Spearman correlation coefficients (*ρ*) between the Bacteria phyla (**A**) and Proteobacteria genus (**B**) and the Halpern database extracted environmental conditions, along the surveyed surface water samples from the Portugal mainland and islands.

Due to the prevalence of Proteobacteria in the surveyed samples, the genus composition of the OTUs detected from this phylum was also analysed in further detail in terms of relative abundance (Figure 2B). At this taxonomic level, a more homogenous taxa distribution was observed compared to the phylum relative abundance across the evaluated samples (Figure 3A). The most abundant genus across the surveyed samples was *Candidatus Pelagibacter*, ranging from 78.6% to 2.84%, with an average relative abundance value across all samples of 48.9%. This relative genus abundance showed a strong inverse correlation (Figure 3B) with almost all the other assessed Proteobacteria genera (except with the *Candidatus Puniceispirillum*, *Erythrobacter* and *Vibrio* genera). Proteobacteria from the genus *Candidatus Puniceispirillum* also showed a relatively high prevalence in some samples (OSD101—Quinta do Lorde, OSD117—Tavira Beach, OSD153—Faro Island, OSD158—São Miguel—II), representing 14–17% of the Proteobacteria abundance. Nevertheless, this genus was also relatively abundant in the remaining samples, with an average relative abundance of 7.7% (maximum = 17.5%, minimum = 0.57%). Similarly to what was found for *Candidatus Pelagibacter*, an inverse correlation with the majority of the surveyed Proteobacteria genera was observed (Figure 3B). Besides the prevalence of these genera, some punctual, high relative abundances of specific genus at specific samples were also evident. The *Vibrio* genus was particularly abundant in the samples collected at Lisbon (OSD107) and Óbidos Lagoon (OSD116). Except for *Alcanivorax*, *Alteromonas* and *Sphingopyxis*, the *Vibrio* genera showed significant inverse correlations with the majority of the surveyed Proteobacteria genera (Figure 4B). Although at a lower extent, the *Luminiphilus* genera also showed high relative abundances in the samples collected at Santa Cruz (OSD115) and Faro Island (OSD153). Faial site samples showed a high prevalence of Proteobacteria from the *Burkholderia* and *Methylococcus* genera. The samples collected at Figueira da Foz (OSD110) showed a high abundance in *Alteromonas* genus Proteobacteria.

Regarding the relationship between the Bacteria phyla’s and Proteobacteria genera’s relative abundance and the abiotic factors of the sampling sites, some significant correlations could be found both at the phylum (Figure 4A) and the genus (Figure 4B) levels. Salinity was the most influencing factor, presenting significant inverse correlations with the relative abundance of each phylum. Proteobacteria’s and Bacteroidetes’ relative abundance was higher in the samples collected near the coast (Figure 4A). This last phylum also showed itself to be positively correlated with the shipping influence. Cyanobacteria’s and Proteobacteria’s relative abundance were found to have an inverse correlation with the site water temperature. Considering the dominance of Proteobacteria over the remaining phyla, the relative abundance of the genus from this phylum was also compared to the site’s abiotic conditions (Figure 4B). *Ruegeria*, *Oceanicola Octadecabacter*, *Rhodobacter*, *Sagittula*, *Loktanella*, *Roseobacter*, *Dinoroseobacter*, *Oceanibulbus*, *Celeribacter* and *Phaeobacter* genera relative abundances showed significant positive correlations with site pollution and shipping influence (wherewith this last also had a strong positive correlation with the genus *Shewanella*). The *Roseovarius*, Pseudomonas, *Burkholderia*, *Marinobacter*, *Methylococcus* and *Labrenzia* genera showed a significant positive correlation with the site distance to the coast, while the *Labrenzia* genus’ (Proteobacteria) relative abundance showed the inverse trend. Analyzing the sites’ Proteobacteria genera abundance and correspondent environmental features (Figure S1), it is possible to observe that the majority of the samples (sites) are located in the lower half of the PCoA biplot, aligned with the majority of the anthropogenic vectors.

### 3.2. InterPro Resistome Signatures

**Figure 5:**
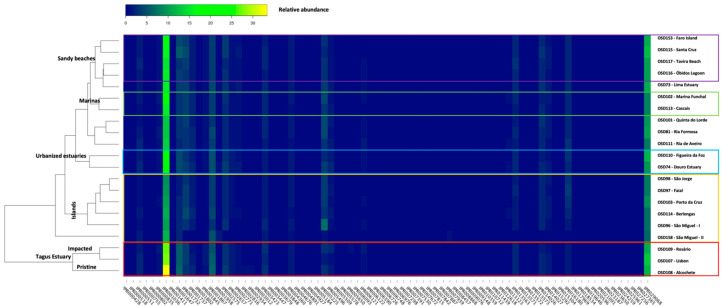
InterPro sequences’ relative abundances along the surveyed surface water samples from the Portugal mainland and islands.

**Figure 6:**
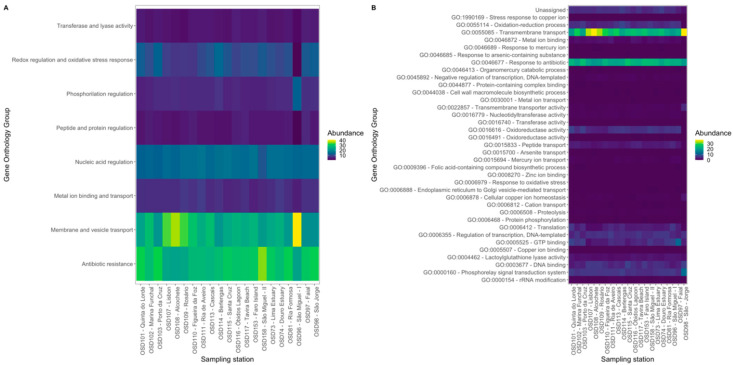
Full (**A**) and merged (**B**) Gene Ontology (GO) terms’ relative abundances along the surveyed surface water samples from the Portugal mainland and islands.

Sequence protein signatures and Gene Ontology terms (GO terms) were assigned by InterPro. This approach allowed for identifying the resistance-associated sequences detected. Considering the resistome, acriflavin resistance protein (IPR00103), the bleomycin resistance protein (IPR029068) and the tetracycline resistance protein TetA/multidrug resistance protein MdtG (IPR001958) (Figure 5) were the most abundant signatures throughout all the surveyed samples. The antibiotic resistance GO terms related InterPro sequences ranged from 23.5 to 35.6% of all the resistome sequences detected in all samples. This high abundance is associated with sequences that present GO terms associated to membrane and vesicle transport (20.3 to 41.2% relative abundance) and nucleic acid regulation (11.4 to 16.1% relative abundance) (Figure 6A,B). Despite these ontologies not being directly associated with resistance (membrane and vesicle transport (e.g., Para-hydroxybenzoic acid efflux pump subunit AaeB/fusaric acid resistance protein, IPR006726) or to nucleic acid regulation (MerR-type HTH domain, IPR000551)), several of the genes analysed present functions that may confer resistance to xenobiotics, including antibiotics. Although with a lower relative abundance, comparative to the sequences related to antibiotic resistance, the InterPro sequences group related with the metal ion’s binding and transport (ranging from 6.3 to 8.0% relative abundance, Figure 6A,B) was also highlighted. Among these, the bile acid sodium symporter/arsenical resistance protein Acr3 (IPR002657), the integral membrane protein TerC (IPR005496) and the heavy metal-associated domain HMA (IPR006121) were the most abundant across the surveyed samples. Some sampling site groups were evident when observing the full InterPro resistance-related sequences as a meta-resistance profile (Figure 5). The first clear separation occurs within the samples collected at the Tagus Estuary. The three resistomes gathered from this transitional system compose a distinct branch, wherein there was also a clear separation of the most pristine site Alcochete (OSD108), located in the Tagus Natural Reserve, from the Rosário (OSD109) and Lisbon (OSD107) sites, near large industrial and urbanized areas, respectively. A second group can be identified by gathering all the samples collected in the Azores archipelago and the resistome of the samples collected in the Berlengas Biosphere reserve (OSD114). The samples of northern and urbanized estuarine systems appear to be grouped according to similarities between their resistomes (OSD110 and OSD74). Another exciting aspect is the grouping resultant from the resistomes gathered in two recreational shipyard sites (OSD102 and OSD113), even though they are highly distant (Portugal mainland and Madeira Island, respectively). Finally, a set of samples collected in the vicinity of sandy beaches was also grouped.

**Figure 7:**
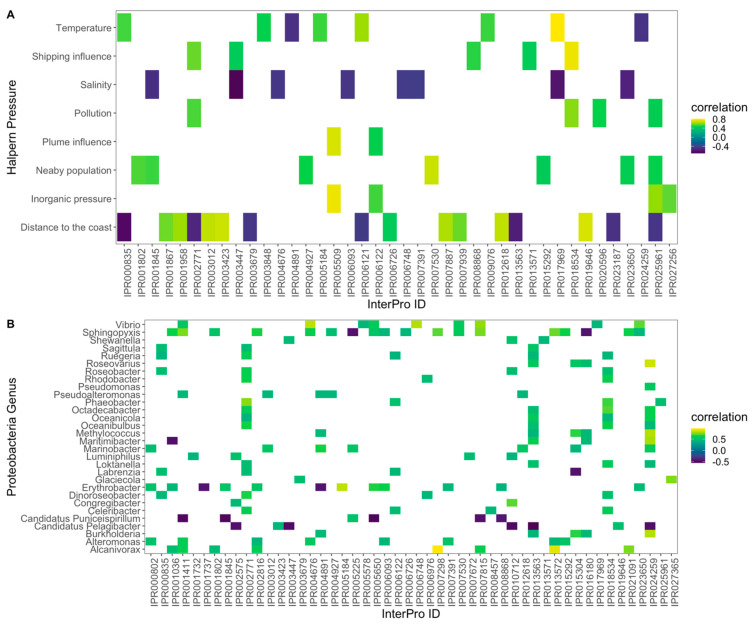
Significant (*p* < 0.05) Spearman correlation coefficients (*ρ*) between the InterPro resistance-related sequences and the Halpern database extracted environmental conditions (**A**) and the relative abundance of the Proteobacteria genus (**B**), along with the surveyed surface water samples from the Portugal mainland and islands.

These differences between resistome profiles are directly related to the previously observed taxonomic composition of the metacommunities and the environmental traits of each sampling site (Figure 7). All the sequences that were assigned through InterPro to resistance features that presented any significant relationship with salinity showed an inverse trend with this abiotic feature, indicating its prevalence in marine environments. Heavy-metal resistance protein (IPR025961) was positively correlated with pollution and inorganic pressures, as well as with the density of nearby population centres and, as expected, was inversely correlated with the distance to the coast. The A-factor biosynthesis hotdog (IPR005509; GO:0046677 response to antibiotic) and heavy metal-associated (IPR006122) domains’ sequences appear highly abundant in the sites with higher plume and inorganic pressure influence. Mercuric transport protein periplasmic component/copper chaperone CopZ (IPR001802), HTH ArsR-type DNA-binding domain (IPR001845), Alkylmercury lyase (IPR004927), Aminoglycoside 6-adenylyltransferase (IPR007530), Transcription regulator YbiH, C-terminal (IPR015292) and Beta-lactamase, class-A active site (IPR023650) sequences’ relative abundance presented a direct and significant correlation with the increasing nearby population density. Multiple antibiotic resistance (MarC)-related (IPR002771), Tetracycline regulation of excision, RteC (IPR018534), Ribosomal RNA adenine methylase transferase and conserved site (IPR020596) sequences, as well as the abovementioned heavy-metal resistance protein (IPR025961) sequence, have their abundance directly and significantly correlated with site pollution. The sites with higher shipping and vessel movement also presented significantly and directly proportional higher relative abundance of the resistance-related sequences, namely, the multiple antibiotic resistance (MarC)-related (IPR002771), FemABX peptidyl transferase (IPR003447), bacterial TniB (IPR008868), transcription regulator QacR, C-terminal (IPR013571) and the Tetracycline regulation of excision, RteC (IPR018534). Several significant correlations were also found between the relative abundance of OTUs from the Proteobacteria phylum genus and the relative abundance of resistance related InterPro sequences (Figure 6B). The genus *Sphingopyxis* showed the highest number of significant correlations with these sequences (18), of which 56% are related to antibiotic resistance and metal ion binding and transport. The relative abundance of these sequences was found to be positively correlated with the abundance of the genus *Sphingopyxis*. The abundance of the genus *Erythrobacter* also showed strong correlations with resistance-related sequences, gathering 58% of the correlated sequences related to antibiotic resistance and metal ion binding and transport. *Vibrio* OTUs’ abundance also showed many significant correlations with resistome-related InterPro sequences (8), with 55% of these being related to antibiotic resistance and metal ion binding and transport. The remaining directly correlated sequences were related to resistance processes linked to protein, nucleic acid, phosphorylation and vesicle regulation and transport. If we analyse in further detail the sequences with a higher number of significant direct correlations with Proteobacteria genus OTUs, the Multiple antibiotic resistance (MarC)-related (IPR002771) shows a strong correlation with 12 Proteobacteria genus OTUs (Celeribacter, Dinoroseobacter, Labrenzia, Loktanella, Oceanibulbus, Oceanicola, Octadecabacter, Phaeobacter, Rhodobacter, Roseobacter, Ruegeria and Sagittula). This was followed by Alkylmercury lyase, a helix-turn-helix domain (IPR024259) sequence relative abundance, which appeared to be significantly and directly correlated with Burkholderia, Loktanella, Marinobacter, Maritimibacter, Methylococcus, Oceanibulbus, Oceanicola, Octadecabacter, Pseudomonas and Roseovarius relative abundances.

## 4. Discussion

Portugal’s mainland and archipelagos (Madeira and Azores) comprise a wide array of coastal and transitional ecosystems in the northeast Atlantic region, varying in typology and degree of human pressure, being, therefore, a natural laboratory to study the ocean microbiome along with a vast array of conditions [26]. It is critical to underpin how these environmental and anthropogenic gradients can shape the microbial communities thriving on these habitats. Microorganisms possess intrinsic genetic traits that allow them to adapt to their abiotic environment by acquiring resistance/tolerance traits [40]. Nevertheless, resistance/tolerance mechanisms sharing the same genetic factors may lead to multidrug resistance/tolerance as well as to co-selection events [41]. Previous works have highlighted that activating metal protective stress responses, such as Cu and Zn, can also trigger and/or protect antibiotics resistance, even without contact with the antimicrobial compounds [22,41,42]. In this context, the degree of human pressure along the Portuguese coast and islands varies significantly with coastal and transitional sites in the vicinity of large metropolitan and industrial areas, oceanic islands and sandy beaches, presenting very different degrees of contamination from anthropogenic sources (e.g., Concepcion et al., 2021).

In taxonomic terms, the samples collected on the Portuguese northeast Atlantic coast waters show an unequivocal prevalence of Bacteria over Archaea, a common feature in marine water samples [43]. This is not unexpected, since these coastal environments are well oxygenated (especially when considering the surface layer), ranging between meso and oligo characteristics (in terms of nutrients, temperature, pH and salinity characteristics) and, thus, lack extreme conditions for the proliferation of extremophile Archaea over Bacteria [43]. Therefore, the present study focused on the abundance of Bacteria. Results show that Portuguese coastal water samples showed a high prevalence of Proteobacteria, Cyanobacteria, Bacteroidetes and Actinobacteria, a very similar profile to those found in other coastal habitats [44]. Proteobacteria and Cyanobacteria were dominant in samples collected across all oceanic provinces during the Tara Oceans expedition [45]. Moreover, our results show an alternation of dominance between Cyanobacteria and Bacteroidetes. In previous works, Bacteroidetes was already the second most abundant group in several sampling sites [46]. Some authors attribute this to different sequencing techniques and the different primers used for amplicon generation, resulting in biased diversity metrics for bacterial communities [47]. Nevertheless, in the present work, all samples were sequenced in the same laboratory, recurring to standardized protocols and uniform primer usage [26], indicating that factors other than primer sets contributed to the observed differences in bacterial phyla abundances.

In terms of the Proteobacteria genus, and despite slight fluctuations in the surveyed samples, *Candidatus Pelagibacter*, *Candidatus Puniceispirillum* and *Vibrio* were prevalent in the analysed samples. *Candidatus Pelagibacter* was the most abundant genus detected in the whole set of analysed water samples. Bacteria of the SAR11 clade, to which *Candidatus Pelagibacter* belongs, are found throughout the world’s oceans and are the prevailing aerobic heterotrophs in marine surface waters [48]. The members of the SAR116 clade, such as *Candidatus Puniceispirillum*, are also known to be highly abundant in marine samples [49]. This supports the high abundance of these Proteobacteria in the water samples from the Portuguese coast. The *Vibrio* genus showed significant inverse correlations with most of the surveyed Proteobacteria genus, particularly abundant in Lisbon (OSD107) and Óbidos Lagoon (OSD116). This genus is known to respond to both temperature and anthropogenic pollution [50]. Although Óbidos Lagoon (OSD116) is not considered heavily disturbed from a human pressure point of view, the nutrient runoff from agricultural fields nearby and its semi-enclosed regime lead to low water renovation rates and increased water temperature [51], thus contributing to the increase in abundance of this genus [50]. As for the Lisbon (OSD107) sampling site, it is located in the vicinity of a densely urbanized area, with all the anthropogenic impacts associated with this [52], also generating the conditions for *Vibrio* proliferation in surface waters [50].

The taxonomical differences among the surveyed sites also lead to changes in the presence and abundance of resistance-related sequences. At this level, the environmental conditions seem to directly and indirectly influence the marine samples’ resistome. If, on one hand, the presence and prevalence of resistance-related genes are known to be stimulated by the contact of the microorganisms with contaminants [25,41], the modulation of the microorganisms’ abundance by these abiotic features will also contribute to the availability of genomes to carry these resistome-related sequences. For example, the presence of heavy metals in the seawater environment can lead to the activation of metal-protective stress responses, which can confer resistance to antibiotics even without contact with the antimicrobial compounds [22,42]. Contact with heavy metals can trigger a higher abundance of sequences coding efflux pumps, providing the organisms ways to excrete these toxic compounds, but also conferring resistance/tolerance to other substances such as antibiotics [22], a mechanism known as cross-resistance [41,53]. Other studies also point towards a similar outcome but with different mechanisms. Silva et al. (2021) showed that a metal-imposed selection of antibiotic-resistant bacteria can also occur. In this work, the exposure to copper increased cefotaxime- and tetracycline-resistant bacteria, while zinc exposure increased the abundance of cefotaxime- and kanamycin-resistant bacteria. These authors suggest a selection of resistant taxa rather than developing cross-resistance genes under contaminant pressure. Whatever the mechanism (or combination of mechanisms) that occurs in marine water bodies under significant anthropogenic pressure, this will unequivocally shape the resistome profile of the bacterial community. This led to the prevalence of resistance-related sequences assigned to gene ontology terms classified into antibiotic resistance, membrane and vesicle transport, nucleic acid regulation and redox regulation and oxidative stress response. A higher number of significant correlations was found between the abundance of resistance-related sequences and the evaluated environmental traits (namely pollution, inorganic pressures and density of nearby population centres) when compared to the number of significant correlations detected between the OTU abundance (either phylum or genus based) and the same environmental conditions, indicating an overall effect of the environmental conditions over the sequence abundance rather than the taxa abundance. These resistome profiles shaped the grouping of the sampling sites. As mentioned above, the first clear separation groups’ sampling sites are located at the Tagus estuary, which are separated into two groups composed of anthropogenically impacted (2) and more pristine areas (1). This separation is not due to differential resistome-related sequences’ presence/absence between sites but to different relative abundances. The Lisboa and Rosário sites (more anthropogenically impacted sites) present coherently higher values of antibiotic and metal resistance-related sequences as well as of several other resistome sequences. This is in line with several previous works where it has been shown that these two areas have higher impacts than the Alcochete area, located within the Tagus Natural Reserve [1,13]. This is also in agreement with the previously observed relationship between, e.g., the heavy-metal resistance protein (IPR025961) and site pollution and inorganic pressure and the density of nearby population centres. Water samples collected at the Rosário sampling site (Tagus estuary, OSD109) and Ria de Aveiro coastal lagoon (OSD111) evidence the higher metal ion binding and transport-related sequences. The individual evaluation shows that, in fact, these two sites share a high prevalence of metal ion binding and transport-related sequences, consistent with the known metal contamination degrees at these sites [1]. Nevertheless, in terms of resistome profile, the location effect prevails over the effect disturbance. Thus, samples collected at the Rosário location appear coupled to the remaining samples collected at the Tagus estuary and are not close to those collected at Ria de Aveiro (a transitional system located ca. 300 km north of the Tagus estuary). Another interesting aspect to consider is the grouping of the samples collected at shipyards and marinas (OSD113—Cascais and OSD102—Marina Funchal). Although the samples collected at these 2 sites have a different number of resistance-related sequences detected, with 80 sequences at OSD113—Cascais and 46 sequences at OSD102—Marina Funchal, the latter site shares its resistance profile completely with OSD113—Cascais, with all the resistance sequences detected in OSD102 being present in OSD113. In fact, and considering the sites located at Madeira Island, the resistome determined for the samples collected at OSD102—Marina Funchal (a marina located in Madeira’s capital Funchal) does not share any resistance-related sequence with OSD103—Porto da Cruz, a site located in the north coast of Madeira and considered to have a low anthropogenic impact. Previous studies have shown the impact of vessels and cruise ships as sources of a wide variety of contaminants such as biocides, pharmaceuticals and heavy metals from ship hulls and discharges [5,54]. The release of these contaminants in such a small semi-enclosed system, such as marinas and shipyards with low water renovation cycles, is one of the main factors shaping the resistome profiles of these sites. Another interesting feature worth noticing is the cluster of the Azorean islands into a single large group with the Berlenga Island site (Biosphere Reserve). Among all the sampled sites, these are probably the less impacted sites (Azorean and Berlenga islands), and, in fact, they show comparatively lower abundances of resistance-related sequences’ relative abundances. The Douro and Mondego estuarine sites (located at the end of the estuarine system and in the vicinity of large metropolitan and industrial areas) were also grouped. Although these sites may share some characteristics with, for example, the Tagus estuary’s Lisbon site, this separation indicates their intrinsic and similar characteristics overlap the potential effect of similar anthropogenic pressures. Finally, a significant group is also formed by the samples collected at sandy beaches with low anthropogenic impact, where two subgroups were evident: one formed by the samples collected at OSD153 (Faro Island) and OSD115 (Santa Cruz) sites, both directly facing the Atlantic open ocean, and a second group formed by the samples collected at OSD117 (Tavira Beach) and OSD116 (Óbidos Lagoon), both collected at beaches within coastal lagoon systems with riverine inputs. Once again, there seems to be a system typology effect, due to their intrinsic characteristics, that, in systems with low disturbance levels, overlap the anthropogenic factors.

Although the carrier organisms of the resistance-related sequences can be diverse, some of the correlations observed indicate some potential and plausible organisms as sources of these sequences. One of the Proteobacteria genera that showed a higher abundance of positive correlations with the abundance of resistome sequences was *Sphingopyxis*. The organisms belonging to this genus are versatile, widely known for their role in environment nutrient cycling, biotechnological practices, intake and metabolism of pesticides and other toxic compounds [55]. The organisms from this genus are also known for developing a multidrug resistance tripartite system to increase their survival chances against specific antimicrobial components in the environment [56]. Additionally, the core genome of species from this genus also presented annotated genes for copper homeostasis, ammonia metabolism, folate and zinc transport, suggesting other possible resistance mechanisms [55]. *Erythrobacter* genus members are also good potential candidates as sources of the resistance-related sequences. *Erythrobacter*’s large pan-genome size indicates that it can acquire foreign genes, which contribute to a more flexible genome and possibly to environmental adaptation [57]. These are probably the two genera whose members most likely contribute, albeit not exclusively, to the shaping of the resistome of the different sampling sites.

On the other hand, sequences such as the multiple antibiotic resistance (MarC)-related (IPR002771) sequence were highly ubiquitous in the surveyed samples, being correlated with 12 different proteobacteria genera OTUs as well as highly correlated with the pollution and shipping influence of the sites. It is fair to assume that this sequence is not only typical to several genera, but it can also respond to anthropogenic pressure. These characteristics’ ubiquity and relationship with the environment’s anthropogenic pressure make it a candidate for a suitable pressure resistome biomarker. Another highly cosmopolitan sequence in terms of carrier genus is the well-known alkylmercury lyase sequence, also known as the *mer*B gene, which encodes an enzyme that degrades the highly toxic methyl-Hg form into lesser toxic ones. A recent study shows that mercury resistance genes are widely distributed in the marine realm [58]. Nevertheless, it is also known that its abundance responds to pollution gradients [59] and, therefore, considering its ubiquity, this resistome sequence can also provide a good candidate biomarker. Overall, despite the identification of these specific sequences, our data suggest that the whole resistome profile can provide more relevant or integrative answers in terms of anthropogenic disturbance of the environment, either as a whole or grouped in gene ontology groups.

## 5. Conclusions

This is a pioneer effort, providing a first picture of the Portuguese transitional and marine realm resistome and evidencing that it is mainly composed of sequences that encode antibiotic and metal resistance traits. Results show the prevalence of Bacteria over Archaea, particularly of Proteobacteria, Cyanobacteria, Bacteroidetes and Actinobacteria, and several taxa (e.g., *Vibrio* genus) were significantly correlated with anthropogenic pollution. From the risk assessment and ecological points of view, these sequences seem to have an excellent relationship with the anthropogenic disturbance of the collection sites, providing good marker candidates for future biomonitoring purposes instead of taxonomic based approaches. Nevertheless, it is essential to bear in mind that the abundance of resistance/tolerance genes in such environments may be attributed to several non-exclusive factors, including (i) co-selection events in response to concentrations of heavy metals, (ii) impacts of human activities, (iii) increase in horizontal gene transfer and co-resistance events by biofilm formation, and other abiotic factors. Another important finding from our data is the need for an efficient sampling program, since site characteristics can overlap the effect of the anthropogenic disturbance when comparing sites with low human-driven impacts. The performance of similar studies with higher sampling replication with additional collection and isolation of bacteria could not only provide more evident insights into the resistome geographical profiles but also allow us to attribute certain antibiotic and metal resistance traits to specific isolates, identifying the key bacterial players for the coastal resistomes.

## Figures and Tables

**Table 1:** Sampling site metadata measured in situ (salinity and temperature) and extracted from the Halpern database [28] (a.u., arbitrary units) and number of prokaryotic sequences correctly assigned.

Site	Number of Sequences	Temperature (°C)	Salinity (PSU)	Distance to Coast (Km)	Inorganic Pressure (a.u.)	Pollution (a.u.)	Plume Influence (a.u.)	Shipping Influence (a.u.)	Nearby Population (a.u.)
OSD101—Quinta do Lorde	107,432	20.50	37.00	0.47	0.46	0.40	0.51	59.13	959.381
OSD102—Marina Funchal	67,004	20.80	36.00	0.40	1.89	0.5	0.81	20.61	4686.554
OSD103—Porto da Cruz	73,592	20.20	37.00	1.69	0.35	0.20	0.41	38.53	1960.618
OSD107—Lisbon	44,340	20.20	30.00	0.05	13.49	0.21	7.26	1043.46	8346.504
OSD108—Alcochete	47,730	20.10	30.00	0.36	2.86	0.21	2.32	1163.10	500.485
OSD109—Rosário	42,947	20.50	30.00	3.82	26.38	0.37	23.72	746.34	5045.171
OSD110—Figueira da Foz	37,659	23.00	22.50	1.13	22.23	0.52	8.36	2217.20	1444.238
OSD111—Ria de Aveiro	89,743	25.20	30.00	0.65	16.19	1.02	6.18	8763.42	941.3195
OSD113—Cascais	112,246	20.20	30.00	0.05	23.35	1.16	8.30	3875.05	3864.33
OSD114—Berlengas	107,131	18.50	33.57	9.22	0.00	0.50	0.00	226.06	0.00
OSD115—Santa Cruz	88,923	20.30	40.00	0.10	2.94	0.22	2.38	1161.79	488.97
OSD116—Óbidos Lagoon	43,828	24.70	22.50	0.74	1.11	0.09	0.86	1239.22	208.93
OSD117—Tavira Beach	81,348	23.64	37.93	0.75	80.71	0.72	206.89	1727.71	1173.97
OSD153—Faro Island	129,914	21.10	34.40	0.51	4.25	0.51	2.54	2168.11	360.03
OSD158—São Miguel—II	152,437	19.20	35.70	33.87	0.00	0.34	0.00	43.57	0.00
OSD73—Lima Estuary	116,182	18.40	32.30	0.58	14.57	1.34	6.15	13,681.47	1176.63
OSD74—Douro Estuary	121,027	20.20	13.75	0.70	10.16	1.52	4.01	15,309.65	438.40
OSD81—Ria Formosa	115,610	22.20	34.30	0.08	4.217	0.49	2.53	2277.45	388.36
OSD96—São Miguel—I	61,474	18.50	35.00	32.81	0.00	0.33	0.00	42.70	0.00
OSD97—Faial	90,089	16.90	35.60	2.13	0.22	0.43	0.99	57.91	432.38
OSD98—São—Jorge	91,817	18.70	35.60	0.86	0.07	0.30	0.74	84.24	106.86

## Data Availability

Raw data deposited at EBI (https://www.ebi.ac.uk/metagenomics/studies/MGYS00000462#overview (accessed on 14 September 2022)).

## Supplementary Materials

The following supporting information can be downloaded at: https://www.mdpi.com/article/10.3390/toxics10100613/s1.
