# Supplementary figures and images for: Unravelling the Portuguese Coastal and Transitional Waters’ Microbial Resistome as a Biomarker of Differential Anthropogenic Impact
Source: Toxics. 2022 Oct 15;10(10):613. doi: 10.3390/toxics10100613 (PMC9612280; doi:10.3390/toxics10100613)

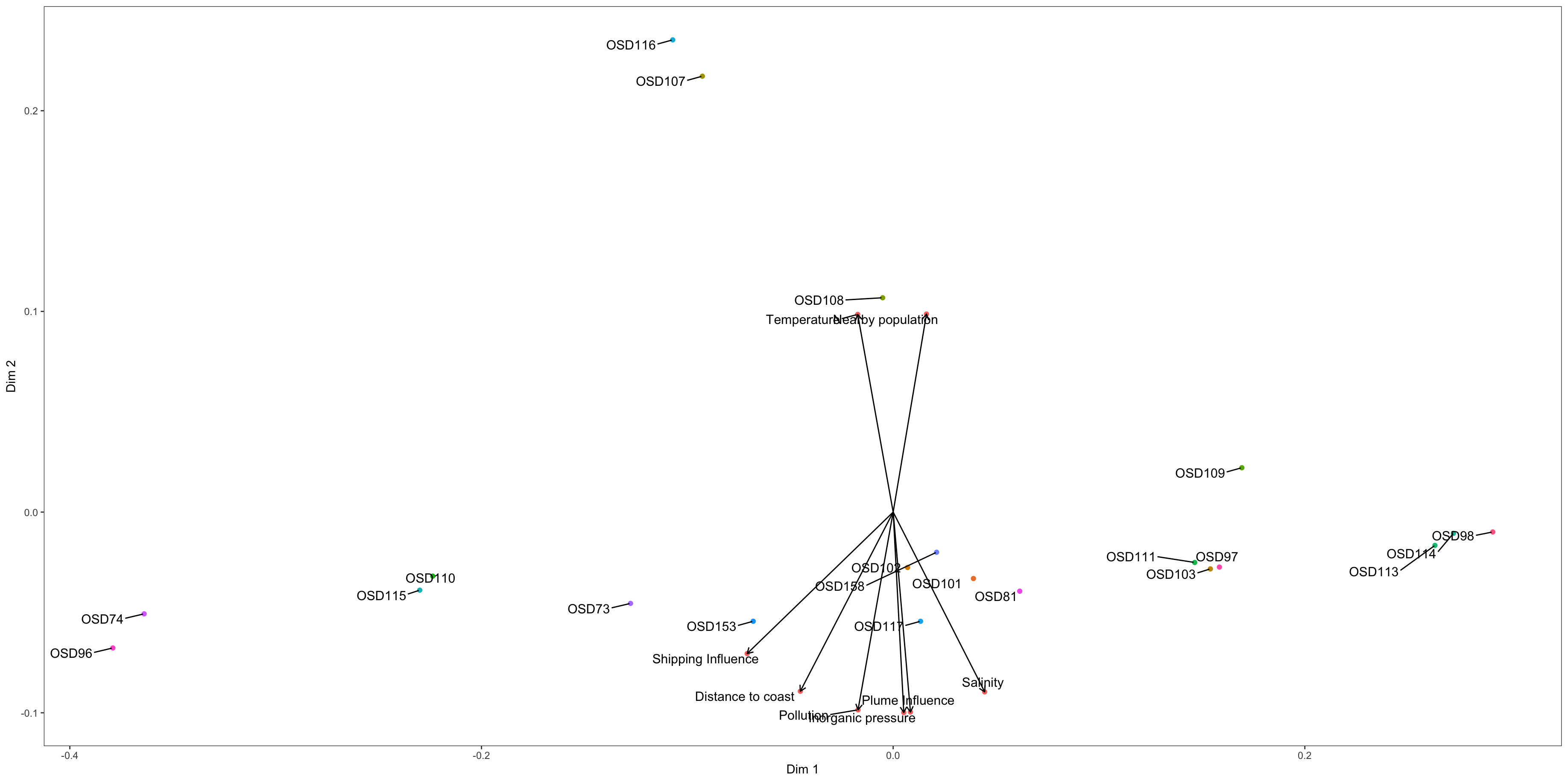

Supplement: Supplementary file 1 [file toxics-10-00613-s001.zip › toxics-1944644-supplementary.png]
